# Supplementary material for: Maternal stress and placental function; ex vivo placental perfusion studying cortisol, cortisone, tryptophan and serotonin
Source: PLoS One. 2020 Jun 3;15(6):e0233979. doi: 10.1371/journal.pone.0233979 (PMC7269612; doi:10.1371/journal.pone.0233979)
Supplement: S1 Table — The three success criteria are defined at different stages of the placental perfusion process: Criteria 1 is a successful cannulation, criteria 2 is a successful preperfusion, and criteria 3 is a successful 6 hour perfusion with added test-substances. (DOCX) [file pone.0233979.s001.docx]

|  | Placentas reaching success criteria (%) |
| --- | --- |
| Placentas received | 48 |
| Criteria 1 (cannulations in system) | 43 (90) |
| Criteria 2 (preperfusion successful: substance added) | 26 (54) |
| Criteria 3 (successful perfusion 6 hours) | 15 (31) |
