# Supplementary material for: Maternal stress and placental function; ex vivo placental perfusion studying cortisol, cortisone, tryptophan and serotonin
Source: PLoS One. 2020 Jun 3;15(6):e0233979. doi: 10.1371/journal.pone.0233979 (PMC7269612; doi:10.1371/journal.pone.0233979)
Supplement: S2 Table — Lifestyle, self-reported health and birth-related outcomes from the women donating the perfused placentas (n = 22). (DOCX) [file pone.0233979.s002.docx]

| Variable | Frequency |
| --- | --- |
| **Maternal age (in years), median [range]** | 35 [24^a^] |
| **Primipartum** |  |
| Yes | 6 |
| No | 16 |
| **Smoking during pregnancy** |  |
| No | 20 |
| Yes | 2 |
| **Alcohol during pregnancy** |  |
| No | 15 |
| Yes | 6 |
| **BMI before pregnancy, kg/m^2^, n=19, mean [range]** | 23 [18-34] |
| **Chronic diseases reported** |  |
| No | 12 |
| Yes | 10 |
| **Gestational complications reported** |  |
| No | 16 |
| Yes | 6 |
| **Medication during pregnancy** |  |
| No | 15 |
| Yes | 7 |
| **Gestational age, median [range]** | 272 [259-290] |
| **Child sex** |  |
| Male | 9 |
| Female | 12 |
| **Anthropometrics, mean [range]** |  |
| Birth weight, g | 3543 [1874^a^] |
| Placenta weight, g | 729 [420^a^] |
| Placenta symmetry, cm | 2.5 [0-11] |

^a^total range to aid anonymization.
