# Supplementary material for: Maternal stress and placental function; ex vivo placental perfusion studying cortisol, cortisone, tryptophan and serotonin
Source: PLoS One. 2020 Jun 3;15(6):e0233979. doi: 10.1371/journal.pone.0233979 (PMC7269612; doi:10.1371/journal.pone.0233979)
Supplement: S3 Table — State stress represented by DASS, PRA, life events and adjusted fetal cortisol exposure (AFCE), trait stress represented by NEO-FFI categories neuroticism and conscientiousness. Cortisol concentration in the fetal system after 30 minutes and from 30 to 300 minutes perfusion represents the rapid initial transfer of cortisol and the steady flow of cortisol through the placenta respectively. Serotonin concentration in the fetal system after 60 minutes and from 60 to 300 minutes perfusion represents the size of the initial peak in serotonin concentration in the fetal system. The concentration values in fetal system are presented as percentage (%) of the added cortisol and serotonin in maternal system (M0 sample). Sum of 5-HIAA concentration in fetal and maternal systems at 6 hours perfusion, presented as % of serotonin in M0 sample. (DOCX) [file pone.0233979.s003.docx]

| nr | DASS  depression | DASS  anxiety | DASS  stress | PRA | Life events | FFI  Neurot. | FFI  Consc. | AFCE | Fetal cortisol ½ h | Δ fetal cortisol ½ to 5 h | Fetal serotonin 1 h | Δ fetal serotonin 1 to 5 h | 5-HIAA  F + M  6 h |
| --- | --- | --- | --- | --- | --- | --- | --- | --- | --- | --- | --- | --- | --- |
| 1 | normal | normal | normal | mild | no | 12 | 40 | 0.017 | 1.8 | -0.25 | 0.17 | -0.16 |  |
| 2 | normal | normal | normal | mild | no | 24 | 26 | 0.014 | 1.1 | 0.19 |  |  |  |
| 3 | normal | normal | normal | mild | no | 23 | 45 | 0.017 | 3.5 | -1.15 | 0.31 | -0.02 | 10.8 |
| 4 | normal | normal | normal | mild | no | 14 | 39 | 0.048 | 1.8 | 2.57 | 0.50 | -0.28 | 2.8^e^ |
| 5 |  |  |  |  | no |  |  | 0.017 | 5.0 | -2.28 | 0.20 | -0.10 | 6.5 |
| 6 | normal | severe | normal | no | no | 17 | 41 | 0.009 | 1.7 | 1.00 | 0.30 | -0.17 | 5.3 |
| 7 | normal | severe | normal | moderate | yes | 32 | 34 | 0.030 | 3.5 | -0.88 | 0.06 | -0.05^c^ | 6.7 |
| 8 | normal | normal | normal | no | no | 8 | 43 | 0.077 | 2.0 | 0.58 | 0.36 | -0.33 | 4.9^e^ |
| 9 | normal | normal | normal | no | no | 7 | 40 | 0.017 | 2.8 | -0.30 | 0.25 | -0.20 | 8.2^f^ |
| 10 | normal | normal | normal | mild | no | 9 | 35 | 0.063 | 3.5 | -1.29 | 0.65 | -0.61 | 5.2 |
| 11 | normal | normal | normal | mild | no | 3 | 42 | 0.019 | 4.8 | -1.90 | 0.22 | -0.21 | 2.8 |
| 12 | normal | normal | normal | mild | no | 9 | 42 | 0.018 | 14.0 | -5.67 | 0.27^b^ | -0.19 | 3.7 |
| 13 | normal | normal | normal | mild | yes | 23 | 27 | 0.040 | 4.1 | 2.09^a^ | 0.13 | 0.12 | 2.9^g^ |
| 14 | normal | normal | normal | mild | yes | 22 | 44 | 0.027 | 3.0 | -0.85 | 2.10 | -1.67 | 7.7 |
| 15 | normal | normal | normal | mild | no | 13 | 36 | 0.027 | 1.5 | 0.40 | 0.54 | -0.28^d^ | 9.8 |
| 16 | normal | normal | normal | mild | no | 22 | 35 | 0.056 | 2.3 | -0.52 | 1.66 | -1.24 | 4.4 |
| 17 | normal | severe | normal | mild | yes | 24 | 35 | 0.009 | 2.5 | -0.82 | 1.82 | -1.05 | 4.3 |
| 18 | normal | normal | normal | moderate | yes | 13 | 37 | 0.019 | 2.3 | 0.11 | 0.23 | -0.22 | 2.2 |
| 19 | normal | normal | normal | mild | no | 19 | 32 | 0.012 | 4.2 | -1.38^a^ | 0.08 | -0.03 | 3.8^g^ |
| 20 | normal | normal | normal | mild | yes | 15 | 34 | 0.033 | 2.4 | -0.80 | 0.12 | -0.10 | 3.6 |
| 21 | normal | normal | normal | moderate | yes | 19 | 48 | 0.025 | 2.5 | -1.51 | 0.10 | -0.05 | 3.6^g^ |
| 22 | normal | normal | normal | mild | yes | 20 | 26 | 0.050 | 2.7 | -0.80 | 0.18 | -0.16 | 4.7 |

Due to missing samples: ^a^cortisol concentration from 4h sample instead of 5h, ^b^serotonin concentration ½h, ^c^serotonin concentration 6h instead of 5h, ^d^serotonin concentration 5½h instead of 5 h, ^e^5-HIAA concentration 5½h, ^f^5-HIAA concentration from fetal system 5h, ^g^5-HIAA concentration 5h.
