# Supplementary material for: Maternal stress and placental function; ex vivo placental perfusion studying cortisol, cortisone, tryptophan and serotonin
Source: PLoS One. 2020 Jun 3;15(6):e0233979. doi: 10.1371/journal.pone.0233979 (PMC7269612; doi:10.1371/journal.pone.0233979)
Supplement: S4 Table — Bivariate correlations between: State stress represented by DASS, PRA, life events and adjusted fetal cortisol exposure (AFCE), trait stress represented by NEO-FFI categories neuroticism and conscientiousness. Cortisol concentration in the fetal system after 30 minutes and from 30 to 300 minutes perfusion represents the rapid initial transfer of cortisol and the steady flow of cortisol through the placenta respectively. Serotonin concentration in the fetal system after 60 minutes and from 60 to 300 minutes perfusion represents the size of the initial peak in serotonin concentration in the fetal system. The concentration values in fetal system are presented as % of the added cortisol and serotonin in maternal system (M0 sample). Sum of 5-HIAA concentration in fetal and maternal systems at 6 hours perfusion, presented as % of serotonin in M0 sample. Correlations were seen between stress variables: life-events, DASS depression, anxiety and stress, and FFI neuroticism. Reverse correlations were seen between related hormone exposure variables: fetal cortisol 30 minutes and Δ fetal cortisol 30–300 minutes, and fetal serotonin 60 minutes and Δ fetal serotonin 60–300 minutes. (DOCX) [file pone.0233979.s004.docx]

| **Correlations** | | | | | | | | | | | | | | |
| --- | --- | --- | --- | --- | --- | --- | --- | --- | --- | --- | --- | --- | --- | --- |
|  | | Life-events | FFI neuroticism | FFI Conscien-tiousness | AFCE | F30-cortisol | ΔF30-F300-cortisol | F60-serotonin | ΔF60-F300-serotonin | 5HIAA | DASS Depression | DASS Anxiety | DASS Stress | PRA |
| Life-events | Pearson Correlation | 1 | ,495^*^ | -,205 | -,004 | -,131 | ,085 | ,131 | -,112 | -,257 | ,757^**^ | ,450^*^ | ,685^**^ | ,486^*^ |
|  | Sig. (2-tailed) |  | ,022 | ,373 | ,985 | ,561 | ,708 | ,570 | ,628 | ,275 | ,000 | ,041 | ,001 | ,025 |
|  | N | 22 | 21 | 21 | 22 | 22 | 22 | 21 | 21 | 20 | 21 | 21 | 21 | 21 |
| FFI neuroticism | Pearson Correlation | ,495^*^ | 1 | -,375 | -,139 | -,229 | ,176 | ,266 | -,162 | ,183 | ,406 | ,620^**^ | ,589^**^ | ,456^*^ |
|  | Sig. (2-tailed) | ,022 |  | ,094 | ,546 | ,318 | ,445 | ,256 | ,496 | ,453 | ,068 | ,003 | ,005 | ,038 |
|  | N | 21 | 21 | 21 | 21 | 21 | 21 | 20 | 20 | 19 | 21 | 21 | 21 | 21 |
| FFI Conscien-tiousness | Pearson Correlation | -,205 | -,375 | 1 | -,116 | ,164 | -,268 | ,080 | -,128 | ,247 | -,150 | -,076 | -,167 | -,216 |
|  | Sig. (2-tailed) | ,373 | ,094 |  | ,618 | ,476 | ,239 | ,737 | ,589 | ,308 | ,516 | ,743 | ,470 | ,348 |
|  | N | 21 | 21 | 21 | 21 | 21 | 21 | 20 | 20 | 19 | 21 | 21 | 21 | 21 |
| AFCE | Pearson Correlation | -,004 | -,139 | -,116 | 1 | -,170 | ,279 | ,105 | -,182 | -,162 | -,112 | -,304 | -,140 | -,234 |
|  | Sig. (2-tailed) | ,985 | ,546 | ,618 |  | ,450 | ,208 | ,649 | ,431 | ,495 | ,629 | ,181 | ,546 | ,307 |
|  | N | 22 | 21 | 21 | 22 | 22 | 22 | 21 | 21 | 20 | 21 | 21 | 21 | 21 |
| F30-cortisol | Pearson Correlation | -,131 | -,229 | ,164 | -,170 | 1 | -,787^**^ | -,153 | ,143 | -,151 | -,118 | -,209 | -,219 | ,222 |
|  | Sig. (2-tailed) | ,561 | ,318 | ,476 | ,450 |  | ,000 | ,508 | ,536 | ,525 | ,609 | ,363 | ,341 | ,334 |
|  | N | 22 | 21 | 21 | 22 | 22 | 22 | 21 | 21 | 20 | 21 | 21 | 21 | 21 |
| ΔF30-F300_cortisol | Pearson Correlation | ,085 | ,176 | -,268 | ,279 | -,787^**^ | 1 | ,029 | ,025 | -,040 | ,023 | ,210 | ,213 | -,221 |
|  | Sig. (2-tailed) | ,708 | ,445 | ,239 | ,208 | ,000 |  | ,901 | ,913 | ,867 | ,922 | ,360 | ,355 | ,336 |
|  | N | 22 | 21 | 21 | 22 | 22 | 22 | 21 | 21 | 20 | 21 | 21 | 21 | 21 |
| F60-serotonin | Pearson Correlation | ,131 | ,266 | ,080 | ,105 | -,153 | ,029 | 1 | -,967^**^ | ,136 | ,164 | ,161 | ,344 | -,280 |
|  | Sig. (2-tailed) | ,570 | ,256 | ,737 | ,649 | ,508 | ,901 |  | ,000 | ,568 | ,488 | ,497 | ,137 | ,232 |
|  | N | 21 | 20 | 20 | 21 | 21 | 21 | 21 | 21 | 20 | 20 | 20 | 20 | 20 |
| ΔF60-F300_serotonin | Pearson Correlation | -,112 | -,162 | -,128 | -,182 | ,143 | ,025 | -,967^**^ | 1 | -,110 | -,092 | -,091 | -,279 | ,291 |
|  | Sig. (2-tailed) | ,628 | ,496 | ,589 | ,431 | ,536 | ,913 | ,000 |  | ,643 | ,699 | ,704 | ,233 | ,214 |
|  | N | 21 | 20 | 20 | 21 | 21 | 21 | 21 | 21 | 20 | 20 | 20 | 20 | 20 |
| 5HIAA | Pearson Correlation | -,257 | ,183 | ,247 | -,162 | -,151 | -,040 | ,136 | -,110 | 1 | -,288 | -,044 | -,022 | -,311 |
|  | Sig. (2-tailed) | ,275 | ,453 | ,308 | ,495 | ,525 | ,867 | ,568 | ,643 |  | ,232 | ,858 | ,930 | ,196 |
|  | N | 20 | 19 | 19 | 20 | 20 | 20 | 20 | 20 | 20 | 19 | 19 | 19 | 19 |
| DASS Depression | Pearson Correlation | ,757^**^ | ,406 | -,150 | -,112 | -,118 | ,023 | ,164 | -,092 | -,288 | 1 | ,633^**^ | ,697^**^ | ,483^*^ |
|  | Sig. (2-tailed) | ,000 | ,068 | ,516 | ,629 | ,609 | ,922 | ,488 | ,699 | ,232 |  | ,002 | ,000 | ,027 |
|  | N | 21 | 21 | 21 | 21 | 21 | 21 | 20 | 20 | 19 | 21 | 21 | 21 | 21 |
| DASS Anxiety | Pearson Correlation | ,450^*^ | ,620^**^ | -,076 | -,304 | -,209 | ,210 | ,161 | -,091 | -,044 | ,633^**^ | 1 | ,639^**^ | ,301 |
|  | Sig. (2-tailed) | ,041 | ,003 | ,743 | ,181 | ,363 | ,360 | ,497 | ,704 | ,858 | ,002 |  | ,002 | ,185 |
|  | N | 21 | 21 | 21 | 21 | 21 | 21 | 20 | 20 | 19 | 21 | 21 | 21 | 21 |
| DASS Stress | Pearson Correlation | ,685^**^ | ,589^**^ | -,167 | -,140 | -,219 | ,213 | ,344 | -,279 | -,022 | ,697^**^ | ,639^**^ | 1 | ,397 |
|  | Sig. (2-tailed) | ,001 | ,005 | ,470 | ,546 | ,341 | ,355 | ,137 | ,233 | ,930 | ,000 | ,002 |  | ,075 |
|  | N | 21 | 21 | 21 | 21 | 21 | 21 | 20 | 20 | 19 | 21 | 21 | 21 | 21 |
| PRA | Pearson Correlation | ,486^*^ | ,456^*^ | -,216 | -,234 | ,222 | -,221 | -,280 | ,291 | -,311 | ,483^*^ | ,301 | ,397 | 1 |
|  | Sig. (2-tailed) | ,025 | ,038 | ,348 | ,307 | ,334 | ,336 | ,232 | ,214 | ,196 | ,027 | ,185 | ,075 |  |
|  | N | 21 | 21 | 21 | 21 | 21 | 21 | 20 | 20 | 19 | 21 | 21 | 21 | 21 |
| *. Correlation is significant at the 0.05 level (2-tailed). | | | | | | | | | | | | | | |
| **. Correlation is significant at the 0.01 level (2-tailed). | | | | | | | | | | | | | | |
